# Bone-Anchored CT Angiography Framework for In Vivo Assessment of Zygomaticus Major Morphology

**DOI:** 10.3390/biomedicines14091906

**Published:** 2026-08-26

**Authors:** Ingrid C. Landfald, George Triantafyllou, Maria Piagkou, Panagiotis Papadopoulos-Manolarakis, Łukasz Olewnik, Michał Podgórski

**Affiliations:** 1 Department of Clinical Anatomy, Mazovian University in Płock, 09-402 Płock, Poland; i.landfald@mazowiecka.edu.pl; 2 “VARIANTIS” Research Laboratory, Mazovian University in Płock, 09-402 Płock, Polandpiagkoumara@gmail.com (M.P.);; 3 VARIA Research Laboratory, Mazovian University in Płock, 09-402 Płock, Poland; 4 Department of Anatomy, School of Medicine, Faculty of Health Sciences, National and Kapodistrian University of Athens, 11527 Athens, Greece; p.papado89@gmail.com; 5 Department of Neurosurgery, General Hospital of Nikaia-Piraeus, 18454 Athens, Greece; 6 Department of Interventional Radiology, Medical University of Lodz, 90-419 Łódź, Poland

**Keywords:** zygomaticus major, CT angiography, facial muscles, anatomical variation, morphometry, imaging anatomy

## Abstract

**Background**: The zygomaticus major (ZMa) is a key mimetic muscle with reported anatomical variability, but in vivo descriptions based on routinely acquired imaging remain limited. **Methods**: This retrospective single-centre study evaluated whether clinically indicated head-and-neck CT angiography (CTA) can support a bone-anchored, consensus-based descriptive assessment of ZMa morphology. Seventy-five CTA examinations were analysed bilaterally, yielding 150 hemifaces from 42 males and 33 females. Multiplanar reconstructions were reviewed using skeletal landmarks. **Results**: ZMa morphology was categorised as Type I (single-bellied), including Type Ia with independent zygomatic origin and Type Ib with proximal fusion with the zygomaticus minor; Type II (double-bellied); or Type III (three-bellied). The ZMa was identifiable in all hemifaces. Type I was observed in 86.7% of hemifaces, including Type Ia in 78.7% and Type Ib in 8.0%; Type II occurred in 12.0% and Type III in 1.3%. Patient-level asymmetry was present in 22.7%. Mean ZMa length was 41.77 ± 6.21 mm, proximal attachment width 5.95 ± 1.36 mm, and distal attachment width 4.39 ± 1.41 mm. These findings suggest that routinely acquired CTA may provide an opportunistic, bone-referenced approach to the descriptive in vivo assessment of visible ZMa morphology and cohort-specific morphometric characteristics. **Conclusions**: CTA should not be considered a reference-standard modality for facial muscle assessment, and further validation using dedicated soft-tissue imaging and formal reliability testing is required.

## 1. Introduction

The zygomaticus major (ZMa) is one of the principal mimetic muscles involved in elevation of the oral commissure and smile formation. It typically arises from the zygomatic bone and courses inferomedially toward the modiolus, where it contributes to the dynamic architecture of the nasolabial region and interacts with the perioral muscular complex. Its close anatomical relationship with the zygomaticus minor (ZMi), orbicularis oculi, levator labii superioris complex and other muscles converging around the oral commissure makes the ZMa relevant not only for descriptive anatomy, but also for imaging interpretation and selected clinical contexts involving the midface [1,2,3,4,5].

Although the ZMa is often presented in standard anatomical descriptions as a single-bellied muscle with a zygomatic origin and modiolus-related insertion, cadaveric studies have shown that its morphology is more variable than this simplified model suggests. Bifid or double-bellied forms have been documented in several anatomical series, with reported prevalence differing according to population, preparation method and terminology [6,7,8]. Additional studies have described variation in the bony attachment of the ZMa on the zygomatic bone, differences in its distal arrangement near the oral commissure and complex relationships with adjacent mimetic muscles [2,9,10,11]. These findings indicate that ZMa morphology cannot always be reduced to a single linear muscular band extending from the zygomatic bone to the modiolus.

A further difficulty is terminological inconsistency. The terms “bifid”, “double”, “double-bellied” and “multibellied” have not always been used in the same way across the anatomical and clinical literature. In some descriptions, a split belly or duplicated distal arrangement is emphasised, whereas in others the relationship between the ZMa and the ZMi or the layered arrangement at the oral commissure is the main point of interest [2,6,7,8]. This distinction is important because a true separation into two or more muscular bellies is not necessarily equivalent to proximal continuity or partial fusion between adjacent mimetic muscles. In particular, distinguishing proximal continuity between the ZMa and ZMi from separation of the ZMa into two or more distinguishable muscular components may help avoid conflating anatomically different configurations under overlapping terms such as “bifid” or “double-bellied”.

In vivo assessment of ZMa morphology remains less developed than cadaveric description. High-resolution ultrasound can visualise superficial facial muscles and allows dynamic assessment, which is particularly useful when muscle mobility, thickness or functional behaviour is clinically relevant [12,13,14]. Ultrasound elastography has also been used to assess mechanical properties of facial muscles, including the ZMa, providing quantitative information that differs from purely morphological imaging [15,16]. MRI offers superior soft-tissue contrast and can be useful for detailed anatomical mapping, whereas three-dimensional craniofacial imaging and surface-based techniques support spatial analysis and skeletal or soft-tissue co-registration in selected contexts [17,18,19,20]. More broadly, advances in segmentation and artificial intelligence have increased interest in automated or semi-automated analysis of craniofacial anatomy, although such approaches remain insufficiently established for routine categorisation of subtle mimetic muscle variants [21,22].

Computed tomography angiography (CTA) is neither a reference-standard nor a dedicated soft-tissue modality for detailed evaluation of facial mimetic muscles. Nevertheless, head-and-neck CTA is frequently performed for clinical indications and often includes the midface within the field of view. Its main potential advantage in this context is not superior soft-tissue contrast, but the availability of consistent osseous landmarks, particularly the zygomatic bone, which may support bone-referenced multiplanar review. Given the superficial course of the ZMa and its bone-referenced relationship to the zygomatic region, clinically acquired CTA may provide incidental anatomical information when image quality, reconstruction parameters and field of view are adequate.

This potential imaging relevance is important because ZMa morphology is considered in several midfacial anatomical and procedural contexts. The muscle is related to superficial fascial layers, facial fat compartments, the parotid duct region and branches of the facial nerve, all of which are relevant to anatomical interpretation of the midface [23,24,25,26,27,28,29,30]. However, CTA-based assessment should not be interpreted as a surgical decision-making tool. Rather, a structured description of visible ZMa morphology may support communication between anatomy and radiology and may generate hypotheses for future studies using dedicated soft-tissue imaging, dynamic assessment or operative correlation.

On this basis, the present study evaluated whether clinically acquired head-and-neck CTA can support a bone-anchored, consensus-based descriptive assessment of ZMa morphology. The aims were to describe an imaging-compatible CTA framework for in vivo assessment of the ZMa, distinguish single-bellied morphology from proximal ZMa-ZMi continuity and double- or three-bellied configurations, report cohort-specific prevalence and morphometric measurements, assess side-related patterns and patient-level asymmetry, and provide a structured reporting template for future validation studies.

## 2. Materials and Methods

### 2.1. Study Design, Ethics and Sample

This was a retrospective, single-centre imaging study based on clinically indicated contrast-enhanced head-and-neck CT angiography (CTA) examinations. CTA examinations were acquired using a 128-slice helical low-dose CT scanner (SOMATOM go.Top, Siemens Healthcare GmbH, Forchheim, Germany). Image analysis was performed using Horos software (version 4.0.1, Horos Project, New York, NY, USA). The study included 75 CTA examinations, analysed bilaterally, yielding 150 hemifaces. The cohort consisted of 42 males and 33 females, with a mean age of 61.6 ± 13.1 years. All examinations were acquired for clinical indications at the General Hospital of Nikaia–Piraeus, Greece. No dedicated facial muscle imaging protocol was used.

A post hoc sensitivity power analysis was conducted to evaluate the adequacy of the sample size for exploratory continuous subgroup comparisons using G*Power statistical software version 3.1.9.7 (Heinrich-Heine-Universität Düsseldorf, Düsseldorf, Germany). With a total sample of 150 hemifaces (84 male and 66 female hemifaces), an alpha error probability of 0.05, and a statistical power of 0.80, the study was sufficiently powered to detect a small-to-medium effect size (Cohen’s d = 0.41) for independent-samples *t*-tests. Additionally, for the primary categorical descriptions, the sample of 150 hemifaces yields a 95% confidence interval with a margin of error of approximately 5.4%.

The study was approved by the Ethics Committee of the General Hospital of Nikaia–Piraeus, Greece (protocol no. 56485; approval date: 13 November 2024), and was conducted in accordance with the Declaration of Helsinki and relevant institutional regulations. Because the study was retrospective and used anonymised clinically acquired CTA data, the requirement for individual informed consent was waived by the Ethics Committee. No identifiable patient information is included in the manuscript or figures.

CTA examinations were included when both midfacial regions were present within the field of view and image quality was sufficient for multiplanar assessment of the zygomaticus major (ZMa). Examinations with visible postoperative changes or surgical distortion involving the zygomatic or perioral region were not included. Unless otherwise stated, categorical counts and morphometric data are reported at the hemiface level; patient-level summaries, including bilateral symmetry and asymmetry, are explicitly labelled.

### 2.2. CTA Acquisition and Reconstruction

CTA examinations were acquired using a SOMATOM go.Top 128-slice helical CT scanner (Siemens, Erlangen, Germany), with patients positioned supine and the head in a neutral position. Iodinated contrast medium, 60 mL, was administered intravenously at a flow rate of 4–4.5 mL/s. Axial, coronal and sagittal multiplanar reconstructions were generated for review, and three-dimensional volume-rendered reconstructions were used for anatomical orientation and figure preparation. Image review and measurements were performed using Horos software (Horos Project, Brooklyn, NY, USA), an open-source imaging platform previously used for segmentation and linear measurement tasks [31].

Acquisition and reconstruction parameters reflected the routine clinical CTA protocol rather than a dedicated facial soft-tissue imaging protocol. Detailed acquisition and reconstruction parameters were not consistently retrievable because of the retrospective clinical nature of the dataset. This limitation was taken into account when interpreting the visibility of small muscle subdivisions and subtle intermuscular planes.

### 2.3. Image Review and Consensus Procedure

Two observers (ICL and GT) independently reviewed all CTA examinations and recorded ZMa morphology and morphometric measurements using predefined bone-anchored criteria. Multiplanar reconstructions were the primary basis for classification and measurement, whereas volume-rendered reconstructions were used as complementary anatomical orientation tools. Disagreements between the two observers were resolved by consensus with senior authors, and the consensus dataset was used for the final descriptive and inferential analyses.

Inter-rater reliability was assessed using Cohen’s kappa (κ) for categorical variables and the Intraclass Correlation Coefficient (ICC) for continuous variables. Cohen’s kappa was used to evaluate agreement between observers for categorical data (types), with a κ value of ≥0.60 considered indicative of at least moderate agreement. The ICC was calculated to assess the consistency of continuous measurements between observers, with values of ≥0.75 considered indicative of good agreement.

### 2.4. CTA-Based Categorisation of ZMa Morphology

ZMa morphology was categorised using a bone-anchored, imaging-compatible framework based on previous anatomical studies documenting bifid, double-bellied, multibellied and variable attachment patterns of the Zma [2,6,7,8,9,10,11]. The zygomatic bone was used as the principal proximal skeletal landmark, and the distal trajectory was followed toward the modiolus region on multiplanar reconstructions.

Type I was defined as a single-bellied ZMa. Within Type I, Subtype Ia was assigned when the ZMa had an independent proximal origin from the zygomatic bone without visible proximal continuity with the zygomaticus minor (ZMi). Subtype Ib was assigned when the ZMa showed proximal continuity or fusion with the ZMi at the origin region. Type II was defined as a double-bellied ZMa, with two distinct bellies separated by an inter-belly cleft and coursing toward the modiolus region. Type III was defined as a three-bellied ZMa with three distinguishable muscular components.

### 2.5. Operational Criteria

A separate belly, corresponding to Type II or Type III morphology, was recorded only when an inter-belly cleft was visible in at least two orthogonal planes and persisted across at least three consecutive slices. Subtype Ib was assigned only when continuous fibre continuity between the ZMa and ZMi in the proximal origin region was visible in at least two planes, without an intervening cleft, across at least three consecutive slices. Borderline cases were conservatively assigned to the simpler category and adjudicated by consensus; therefore, uncertain cases of proximal ZMa-ZMi continuity were classified as Type Ia, and uncertain cases of belly separation were classified as Type I rather than Type II.

### 2.6. Morphometric Measurements

Morphometric assessment included ZMa length, proximal attachment width and distal attachment width. Measurements were performed on multiplanar reconstructions aligned as closely as possible with the course of the muscle. ZMa length was measured from the proximal origin on the zygomatic bone to the distal attachment region at or near the modiolus. Proximal attachment width was measured as the maximum transverse width of the proximal ZMa attachment footprint on the zygomatic bone. Distal attachment width was measured as the maximum transverse width of the distal attachment region near the modiolus. Measurements were recorded in millimetres. In double- or three-bellied variants, ZMa length was measured from the proximal origin to the distal attachment region of the visible ZMa complex rather than as separate belly-specific lengths.

### 2.7. Statistical Analysis

Statistical analysis was performed using IBM SPSS Statistics, version 29 (IBM Corp., Armonk, NY, USA). Results are presented as mean ± standard deviation (SD), unless otherwise stated. Because ZMa morphology and morphometric measurements were assigned separately to each side, the primary descriptive unit was the hemiface. Nominal variables from unpaired observations were compared using the chi-square test, whereas McNemar’s test was used for paired observations where applicable. The Shapiro–Wilk test was used to assess normality. For continuous data, independent-samples *t*-tests were applied when data were normally distributed, and the Mann–Whitney U test was used otherwise. Correlations between morphometric parameters were assessed using Spearman’s rank correlation coefficient. To account for intra-subject dependency arising from bilateral hemiface assessments, mixed-effects models were utilised. Continuous morphometric data (ZMa length, proximal, and distal attachment widths) were analysed using linear mixed-effects models (LMM) with the patient designated as a random effect. For the comparison of categorical morphological variables, generalised estimating equations (GEE) were applied. A *p*-value < 0.05 was considered statistically significant.

## 3. Results

### 3.1. CTA Visibility and Morphological Distribution

The ZMa could be delineated on CTA in all analysed hemifaces (150/150; 100%). Cohen’s κ for the identification and ZMa classification was 1.000, demonstrating complete agreement between the two researchers for the categorical variables. The ICC for the continuous morphometric variables (length and width) ranged between 0.911 and 0.967, indicating excellent consistency.

Using the predefined bone-anchored criteria in the consensus dataset, ZMa morphology was categorised into Type I, Type II and Type III, with Type I further divided into Subtypes Ia and Ib.

**Figure 1:**
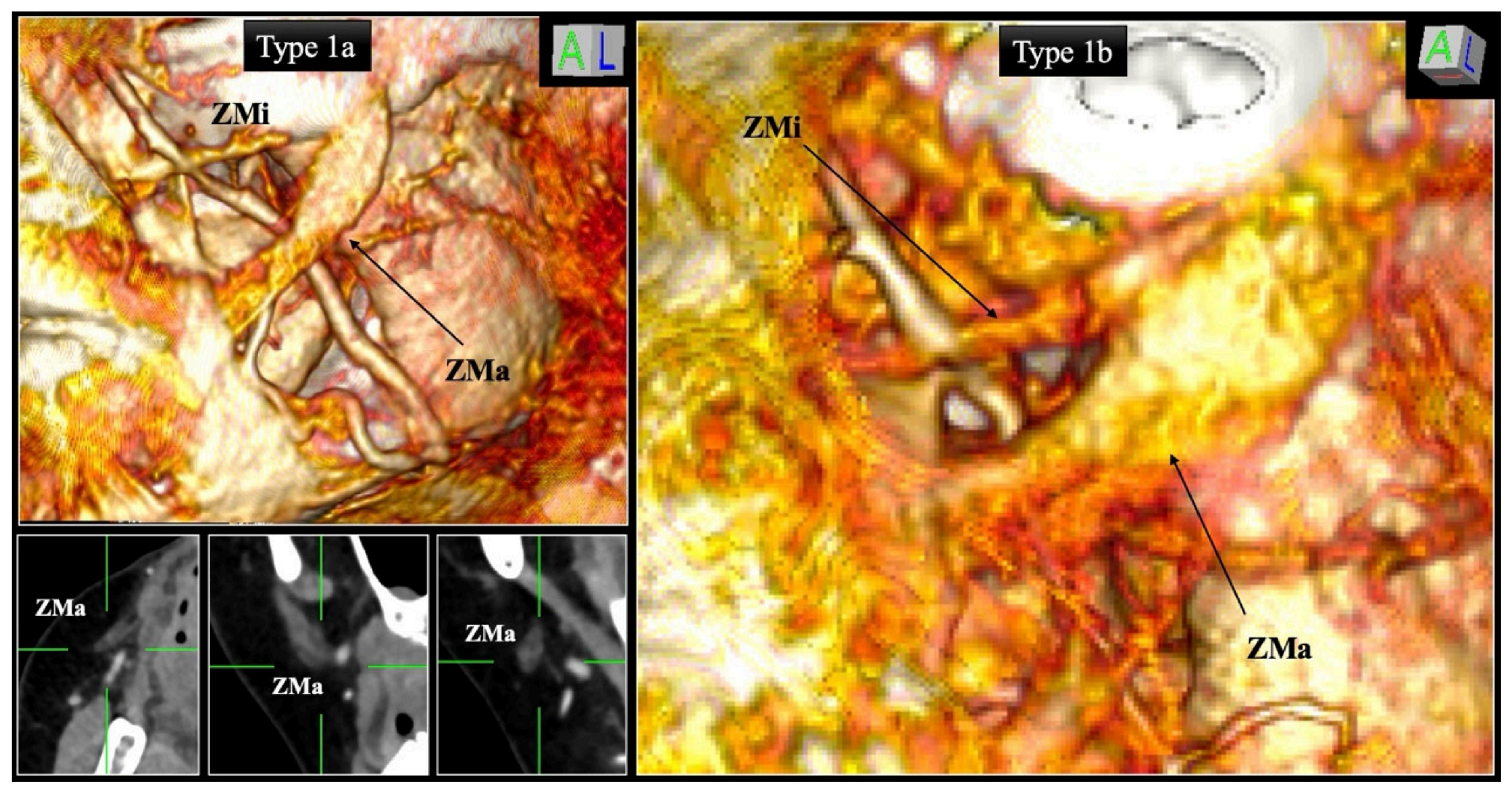
Computed tomography angiography (CTA)-based bone-anchored assessment of zygomaticus major (ZMa) morphology: Type Ia and Type Ib. (**Upper row**) Three-dimensional volume-rendered reconstructions. (**Left**) Type Ia, showing an independent proximal origin of the ZMa from the zygomatic bone and an inferomedial course toward the modiolus region. (**Right**) Type Ib, showing proximal continuity or fusion between the ZMa and the zygomaticus minor (ZMi) at the origin region. (**Lower row**) Multiplanar CTA reconstructions illustrating bone-referenced assessment and morphometric landmarks of the ZMa, including the proximal origin and distal attachment region. Abbreviations: ZMa, zygomaticus major; ZMi, zygomaticus minor.

Type I, corresponding to a single-bellied ZMa, was the most common morphology and was observed in 130/150 hemifaces (86.7%) (Figure 1; Table 1). Within this group, Type Ia, defined by an independent zygomatic origin without visible proximal continuity with the ZMi, was identified in 118/150 hemifaces (78.7%). Type Ib, defined by proximal continuity or fusion between the ZMa and ZMi, was identified in 12/150 hemifaces (8.0%) (Figure 1; Table 1).

**Figure 2:**
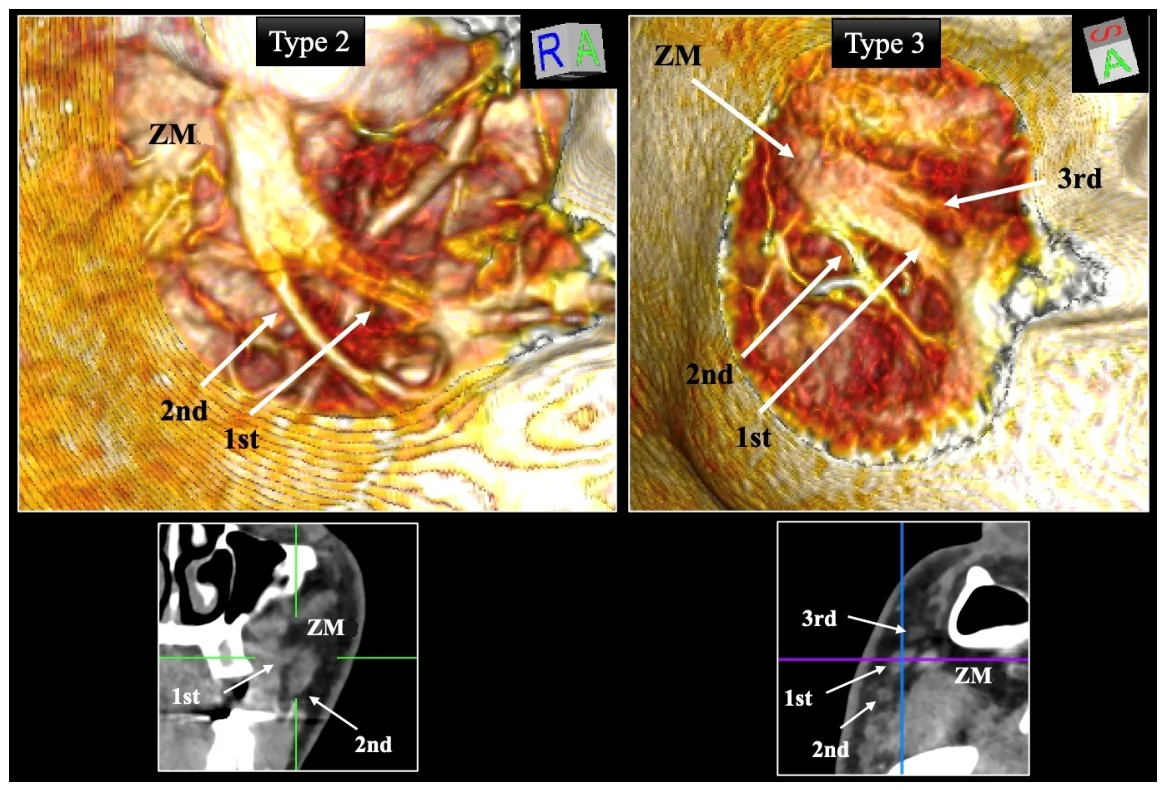
Computed tomography angiography (CTA)-based bone-anchored assessment of multibellied zygomaticus major (ZMa) morphology: Types II and III. (**Upper row**) Three-dimensional volume-rendered reconstructions. (**Left**) Type II, showing a double-bellied ZMa configuration with two distinct muscular components (1st and 2nd) coursing toward the modiolus region. (**Right**) Type III, showing a three-bellied ZMa configuration with three distinguishable muscular components (1st, 2nd, 3rd). (**Lower row**) Multiplanar CTA reconstructions illustrating visible inter-belly separation and the relationship of the muscular components to skeletal reference points. Abbreviation: ZM, zygomaticus major.

Type II, corresponding to a double-bellied ZMa, was present in 18/150 hemifaces (12.0%) (Figure 2; Table 1). Type III, corresponding to a three-bellied ZMa, was rare and was observed in 2/150 hemifaces (1.3%) (Figure 2; Table 1).

### 3.2. Side- and Sex-Related Distribution

The distribution of ZMa morphological categories did not differ significantly between left and right sides (*p* = 0.727; Table 1). At the patient level, bilateral single-bellied morphology was observed in 52/75 patients (69.3%), whereas bilateral double-bellied morphology was observed in 6/75 patients (8.0%). Side-to-side asymmetry was present in 17/75 patients (22.7%). The distribution of ZMa morphology did not differ significantly between males and females (*p* = 0.171; Table 1).

### 3.3. Morphometric Findings

Mean ZMa length was 41.77 ± 6.21 mm. Mean proximal attachment width was 5.95 ± 1.36 mm, and mean distal attachment width was 4.39 ± 1.41 mm (Table 2). In exploratory sex-related comparisons, males showed greater ZMa length than females (43.40 ± 4.45 mm vs. 39.71 ± 5.61 mm; *p* = 0.045) and greater distal attachment width (4.70 ± 1.04 mm vs. 3.74 ± 1.18 mm; *p* = 0.045) and proximal attachment width (*p* = 0.037) (Table 2). In the performed Spearman analysis, the distal and proximal attachment width only showed a positive association (rho = 0.515; *p* = 0.010).

## 4. Discussion

This study describes a bone-anchored, CTA-based framework for incidental in vivo assessment of zygomaticus major (ZMa) morphology in a retrospective clinical imaging cohort. Using predefined multiplanar criteria applied to a consensus dataset, ZMa morphology was categorised into Type I, Type II and Type III, with Type I further subdivided into Type Ia and Type Ib according to the presence or absence of proximal continuity with the zygomaticus minor (ZMi). The analyses were performed on clinically indicated head-and-neck CTA examinations rather than on dedicated facial soft-tissue imaging. Among 150 hemifaces, Type I was the most common morphology, followed by Type II, whereas Type III was rare. Patient-level side-to-side asymmetry was present in approximately one-fifth of individuals. The study also provides cohort-specific morphometric values for ZMa length and proximal and distal attachment widths. These findings support the feasibility of structured, bone-referenced description of visible ZMa morphology on CTA, while requiring further validation using dedicated soft-tissue imaging and formal reliability testing.

The principal contribution of this work is not to establish CTA as the modality of choice for facial mimetic muscle assessment, but to demonstrate that routinely acquired head-and-neck CTA may contain usable anatomical information when the midface is included in the field of view and image quality is sufficient. By anchoring the assessment to the zygomatic bone and following the distal course toward the modiolus region, the present CTA-based descriptive framework provides an imaging-compatible vocabulary for describing ZMa morphology in vivo. The reported measurements should be interpreted as cohort-specific descriptive values rather than normative population reference values, particularly because the cohort consisted of older clinically imaged patients rather than healthy volunteers.

A developmental background may help explain why variability of the ZMa and adjacent mimetic muscles is anatomically plausible. Muscles of facial expression derive from cranial mesoderm associated with the pharyngeal arches, particularly the second pharyngeal arch, and follow developmental programmes that differ from somite-derived limb and trunk musculature [32,33,34,35,36,37]. Cranial neural crest interactions and molecular pathways involved in craniofacial patterning influence the spatial organisation of facial soft tissues. Developmental disturbances involving pharyngeal arch patterning and craniofacial morphogenesis, including conditions associated with TBX1, PAX3 and neural crest-related pathways, illustrate the broader developmental sensitivity of this region [38,39,40,41,42]. However, the present CTA study did not investigate developmental mechanisms directly. Therefore, embryological considerations should be regarded as a biologically plausible anatomical context rather than as evidence explaining the specific frequency of Type Ib or multibellied configurations observed in this cohort.

Previous cadaveric studies have documented that the ZMa is not invariably a simple single-bellied structure. Pessa et al. [7] described double or bifid ZMa morphology and linked this configuration to cheek dimpling, while Hu et al. [6] provided additional anatomical evidence for bifid ZMa forms. Shim et al. [2] focused on the distal arrangement of the ZMa at the corner of the mouth, highlighting the complexity of the modiolus region and its muscular interconnections. Other anatomical studies have described variation in the midfacial musculature, bony attachment of the ZMa on the zygomatic bone and relationships with adjacent structures such as the orbicularis oculi and parotid duct [9,10,11]. A meta-analysis by Phan and Onggo [8] further demonstrated that reported prevalence of bifid ZMa varies across studies, reflecting heterogeneity in definitions, populations and methods. More broadly, recent anatomical and interdisciplinary reviews have emphasised that variability of the zygomaticus muscles remains clinically relevant but inconsistently classified across the anatomical, imaging and procedural literature [43,44].

The principal anatomical rationale for distinguishing Type Ia from Type Ib is to separate two morphologically different phenomena that may otherwise be conflated in existing terminology. Type Ia represents a single-bellied ZMa with an independent zygomatic origin, whereas Type Ib also remains single-bellied but demonstrates proximal continuity or fusion with the ZMi. This differs conceptually from Types II and III, in which the defining feature is separation of the ZMa into two or three distinguishable muscular components. Previous anatomical studies have primarily described bifid or double-bellied ZMa morphology, distal insertional arrangements, and variation in the bony attachment of the muscle (Pessa et al., 1998 [7]; Hu et al., 2008 [6]; Shim et al., 2008 [2]; Sarilita et al., 2021 [11]). The proposed Ia/Ib distinction therefore specifically separates intermuscular ZMa–ZMi continuity from multibellied ZMa morphology. Anatomically, this distinction may improve consistency of description across cadaveric and imaging studies and reduce terminological ambiguity when proximal ZMa–ZMi continuity is encountered. From an imaging perspective, it may also provide a more precise description of the proximal zygomaticus complex when comparing sides or findings across studies. Its direct functional and clinical significance, however, remains to be established and should not be inferred from the present retrospective imaging data. The relationship between the present CTA-based classification and selected previous anatomical studies is summarised in Table 3.

CTA is not a reference-standard modality for facial mimetic muscle imaging and should be regarded as an opportunistic imaging approach in this context. Its principal advantage for the present application is the availability of consistent osseous landmarks, particularly the zygomatic bone, which can support bone-referenced multiplanar assessment. However, limited soft-tissue contrast, partial-volume effects, reconstruction parameters and artefacts remain important constraints. High-resolution ultrasound is better suited for dynamic assessment of superficial facial muscles (Sauer et al. [12] Volk et al. [13]; Youn et al. [14]), whereas MRI provides superior soft-tissue contrast for detailed anatomical mapping (Stephan et al. [19]). Accordingly, the present CTA-based approach should be considered complementary to, rather than a replacement for, dedicated soft-tissue imaging.

The potential clinical relevance of the present work lies primarily in structured anatomical description rather than direct procedural recommendation. The ZMa is closely related to superficial fascial layers, facial fat compartments, the parotid duct region and branches of the facial nerve, all of which contribute to the complex layered anatomy of the midface (Cotofana and Lachman, [23]; Gupta et al., [24]; Hwang, [25]). Variability in ZMa morphology may therefore be relevant when interpreting regional midfacial anatomy. However, the present study did not evaluate surgical findings, injectable procedures, facial nerve course or operative outcomes, and the proposed classification should not be interpreted as a surgical decision-making algorithm.

The patient-level asymmetry observed in this cohort should be interpreted primarily as an anatomical imaging finding. Previous anatomical work has proposed a relationship between double or bifid ZMa morphology and cheek dimpling (Pessa et al., [7]). However, the present retrospective CTA dataset did not include standardised assessments of smiling, facial expression, muscle activation or cheek dimpling. Consequently, direct correlations between ZMa morphology and functional or phenotypic facial characteristics could not be performed, and the observed morphological asymmetry should not be interpreted as evidence of functional asymmetry. Prospective studies incorporating standardised dynamic facial assessment are needed to determine whether specific ZMa morphological patterns are associated with smiling, facial expression or cheek dimpling. This study has several strengths. It represents an in vivo CTA-based series focused specifically on ZMa morphology, rather than relying exclusively on cadaveric observations. The use of skeletal landmarks provides a consistent anatomical reference for reviewing the proximal origin and distal trajectory of the muscle. The framework combines categorical morphology with quantitative morphometric measurements, including length and proximal and distal attachment widths. The study also separates hemiface-level distributions from patient-level bilateral patterns, including asymmetry. Finally, by presenting operational criteria for Types I-III and Subtypes Ia-Ib, the study provides a transparent starting point for future observer-reliability studies and multimodal validation. In this context, Table 4 provides a structured reporting template for documenting visible ZMa morphology and morphometric measurements on CTA, while Table 5 summarises imaging hallmarks and interpretive pitfalls. These tables should be regarded as descriptive aids for future validation rather than as validated clinical checklists.

Several limitations must be acknowledged. First, CTA is neither a reference-standard nor a dedicated facial soft-tissue modality. Delineation of the ZMa may be influenced by partial-volume effects, artefacts and routine reconstruction choices rather than by optimised soft-tissue contrast. Second, detailed acquisition and reconstruction parameters were not consistently retrievable because of the retrospective clinical nature of the dataset, which limits assessment of how protocol variability may have influenced detectability of subtle intermuscular planes. Third, no MRI or ultrasound validation was performed. This is particularly relevant for Type Ib and Type III, where subtle proximal continuity or small inter-belly clefts may be difficult to confirm on CTA alone. Fourth, the study was single-centre and included an older clinically imaged cohort, limiting generalisability to younger individuals, healthy volunteers or other populations. Fifth, side- and sex-related comparisons should be interpreted as exploratory, particularly because the primary descriptive unit was the hemiface and the study was not designed primarily to evaluate sex effects or population-level morphometric norms. Finally, functional and phenotypic data on smiling, facial expression and cheek dimpling were not available, precluding direct morphology–function correlations.

Future studies should validate the proposed CTA-based classification prospectively, across multiple centres, and against dedicated soft-tissue imaging. Future prospective studies should incorporate standardised dynamic facial assessment to determine whether ZMa morphological patterns are associated with smiling, facial expression, cheek dimpling or clinically relevant asymmetry. Such studies would clarify whether the present consensus-based CTA framework can evolve from a descriptive imaging approach into a validated multimodal anatomical reporting system.

## 5. Conclusions

This study describes a bone-anchored, CTA-based approach for opportunistic in vivo assessment of visible zygomaticus major (ZMa) morphology on routinely acquired head-and-neck CTA. Using predefined skeletal landmarks, ZMa morphology was categorised into Types I-III, including Subtype Ib, defined by proximal continuity with the zygomaticus minor. In this older, clinically imaged cohort, no significant side-related differences were observed, patient-level side-to-side asymmetry occurred in 22.7% of patients, and exploratory sex-related comparisons suggested differences in selected morphometric parameters. The accompanying measurements provide cohort-specific quantitative anchors for structured radiological description and hypothesis generation. Further multicentre validation using dedicated soft-tissue imaging, particularly MRI and measurement reliability testing, is required.

## Figures and Tables

**Table 1:** Hemiface-level distribution of zygomaticus major (ZMa) morphological categories by side and sex.

ZMa Morphology	Total (*n* = 150)	Left (*n* = 75)	Right (*n* = 75)	Males (*n* = 84)	Females (*n* = 66)
Type I (single-bellied)	130 (86.7%)	67 (89.3%)	63 (84.0%)	73 (87.1%)	57 (86.2%)
Subtype Ia (independent zygomatic origin)	118 (78.7%)	62 (82.7%)	56 (74.7%)	64 (76.5%)	54 (81.5%)
Subtype Ib (proximal continuity/fusion with ZMi)	12 (8.0%)	5 (6.7%)	7 (9.3%)	9 (10.6%)	3 (4.6%)
Type II (double-bellied)	18 (12.0%)	8 (10.7%)	10 (13.3%)	10 (11.8%)	8 (12.3%)
Type III (three-bellied)	2 (1.3%)	0 (0.0%)	2 (2.7%)	1 (1.1%)	1 (1.5%)

Overall comparison by side: *p* = 0.727. Overall comparison by sex: *p* = 0.171. ZMi, zygomaticus minor. *p*-values for overall comparisons by side and sex were derived using generalised estimating equations (GEE) to account for intra-subject dependency.

**Table 2:** Hemiface-level morphometric measurements of the zygomaticus major (ZMa) by side and sex.

ZMa Morphometry	Total (*n* = 150)	Left (*n* = 75)	Right (*n* = 75)	*p*-Value (Side)	Males (*n* = 84)	Females (*n* = 66)	*p*-Value (Sex)
Proximal attachment (PA) width, mm	5.95 (1.36)	5.90 (1.36)	6.03 (0.97)	0.766	6.18 (1.21)	5.59 (1.50)	0.037 *
Muscle length, mm	41.77 (6.21)	41.64 (5.31)	41.94 (7.06)	0.895	43.40 (4.45)	39.71 (5.61)	0.045 *
Distal attachment (DA) width, mm	4.39 (1.41)	4.24 (1.19)	4.60 (1.41)	0.622	4.70 (1.04)	3.74 (1.18)	0.045 *

Values are presented as mean (standard deviation). PA, proximal attachment; DA, distal attachment. *p*-values for side- and sex-related comparisons were derived using linear mixed-effects models (LMM) with the patient designated as a random effect. Side- and sex-related comparisons should be interpreted as exploratory. * *p* < 0.05.

**Table 3:** Comparative overview of selected zygomaticus major (ZMa) variation frameworks and anatomical studies.

Source	Modality/Setting	Sample Size	Classified or Mapped Features	Key Contribution	Main Limitations for Standardisation
Present study	CTA; clinical patients	150 hemifaces (75 patients)	Types I–III; Type Ib defined as proximal ZMa–ZMi continuity/fusion; ZMa length and proximal/distal attachment widths	Provides an operational in vivo imaging framework that distinguishes proximal ZMa–ZMi continuity (Type Ib) from multibellied ZMa morphology and incorporates bone-referenced morphometry.	Single-centre retrospective CTA cohort; consensus-based assessment; limited soft-tissue contrast; no independent soft-tissue validation.
Pessa et al., [7]	Cadaveric	50 hemifacial dissections	Double/bifid ZMa morphology; relationship with cheek dimpling	Documented double/bifid ZMa morphology and proposed a mechanism linking the bifid configuration with cheek dimpling.	Ex vivo design; terminology not standardised for imaging; no operational in vivo imaging criteria.
Hu et al., [6]	Cadaveric	70 hemifacial dissections	Bifid ZMa morphology	Provided cadaveric evidence of bifid ZMa configurations and their insertional pattern.	Ex vivo design; limited translation to an operational in vivo imaging classification.
Shim et al., [2]	Cadaveric	70 embalmed cadaver specimens	Distal insertional arrangement at the oral commissure	Detailed the arrangement and attachment patterns of ZMa fibres at the modiolus and oral commissure.	Focused on distal insertion rather than an origin-to-insertion morphological imaging framework.
Sarilita et al., [11]	Cadaveric; Thiel-embalmed	26 bodies (52 facial dissections)	Bony origin/attachment site on the zygomatic bone; relationship to the masseter	Defined a palpable bony landmark for the ZMa origin on the zygomatic bone.	Ex vivo design; focused primarily on the proximal attachment site rather than complete ZMa morphology.
Elvan et al., [9]	Cadaveric	15 cadaver heads (29 sides)	ZMa morphology and relationships with orbicularis oculi and parotid duct	Provided morphological and topographic data on ZMa and adjacent midfacial structures.	Ex vivo design; not an operational classification focused on ZMa belly number or proximal ZMa–ZMi continuity.
Phan and Onggo, [8]	Meta-analysis of cadaveric studies	7 studies	Prevalence of bifid ZMa morphology across population groups	Synthesised the prevalence of bifid ZMa morphology across cadaveric populations.	Heterogeneous definitions and methods across included studies; no operational in vivo imaging framework.

**Table 4:** Proposed structured CTA reporting template for visible zygomaticus major (ZMa) morphology.

Item	What to Report	Operational CTA Descriptor	Interpretive Note
1. Morphological category	Type Ia, Type Ib, Type II or Type III	Apply bone-anchored criteria using the zygomatic bone as the proximal landmark and the modiolus region as the distal reference. Distinguish an independent single-bellied ZMa from proximal ZMa-ZMi continuity and from separation into two or more distinguishable muscular components.	Report the side assessed and the morphological category.
2. Quantitative measurements	ZMa length, proximal attachment width and distal attachment width	Record measurements on multiplanar CTA reconstructions aligned as closely as possible with the course of the muscle.	Interpret measurements as cohort-specific descriptive values rather than normative reference values.
3. Laterality and asymmetry	Left/right category and bilateral pattern	Report whether the same morphological category is present bilaterally or whether patient-level asymmetry is present.	Interpret asymmetry as an anatomical imaging finding unless supported by functional correlation.

**Table 5:** CTA hallmarks, potential interpretive pitfalls and anatomical value of the proposed zygomaticus major (ZMa) morphological categories.

Type	CTA Hallmarks	Potential Interpretive Pitfalls	Anatomical and Interpretive Value
Type Ia	Single-bellied ZMa with an independent proximal origin from the zygomatic bone and course toward the modiolus region.	Under-recognition of subtle proximal continuity with the ZMi or minor side-to-side differences.	Provides an operational reference single-bellied configuration against which proximal continuity and multibellied morphology can be distinguished.
Type Ib	Single-bellied ZMa with proximal continuity or fusion with the zygomaticus minor (ZMi) at the origin region.	Misclassification as Type Ia; overcalling simple overlap as continuity; confusing ZMa-ZMi continuity with an additional ZMa belly.	Separates intermuscular ZMa-ZMi continuity from multibellied ZMa morphology, thereby reducing terminological ambiguity.
Type II	Double-bellied ZMa with two distinguishable muscular components separated by a visible inter-belly cleft.	Confusing partial clefting, partial-volume effects or artefact with a true double-bellied configuration.	Represents separation of the ZMa into two muscular components and provides an imaging-compatible descriptor for bifid/double-bellied morphology described in previous anatomical studies (Pessa et al. [7]; Hu et al. [6]).
Type III	Three-bellied ZMa with three distinguishable muscular components.	Underestimating complex morphology or overinterpreting artefactual planes as separate bellies.	Extends the framework to rare complex multibellied configurations while maintaining the same operational criterion of visible inter-belly separation.

Note. These categories are intended as anatomical and imaging-reporting descriptors and should not be interpreted as validated clinical risk categories or procedural recommendations.

## Data Availability

The original contributions presented in this study are included in the article. Further inquiries can be directed to the corresponding author.
